# Histopathological Differential Diagnosis of Meningoencephalitis in Cetaceans: Morbillivirus, Herpesvirus, *Toxoplasma gondii, Brucella* sp., and *Nasitrema* sp.

**DOI:** 10.3389/fvets.2020.00650

**Published:** 2020-09-30

**Authors:** Eva Sierra, Antonio Fernández, Idaira Felipe-Jiménez, Daniele Zucca, Josué Díaz-Delgado, Raquel Puig-Lozano, Nakita Câmara, Francesco Consoli, Pablo Díaz-Santana, Cristian Suárez-Santana, Manuel Arbelo

**Affiliations:** ^1^Veterinary Histology and Pathology, Institute of Animal Health and Food Safety, Veterinary School, University of Las Palmas de Gran Canaria, Las Palmas de Gran Canaria, Spain; ^2^Texas A&M Veterinary Medical Diagnostic Laboratory, College Station, TX, United States; ^3^Department of Neuroscience, Imaging and Clinical Sciences, University G. D'Annunzio, Chieti, Italy

**Keywords:** meningoencephalitis, cetaceans, morbillivirus, herpesvirus, *Brucella* sp., *Toxoplasma gondii*, *Nasitrema sp*.

## Abstract

Infectious and inflammatory processes are among the most common causes of central nervous system involvement in stranded cetaceans. Meningitis and encephalitis are among the leading known natural causes of death in stranded cetaceans and may be caused by a wide range of pathogens. This study describes histopathological findings in post-mortem brain tissue specimens from stranded cetaceans associated with five relevant infectious agents: viruses [Cetacean Morbillivirus (CeMV) and Herpesvirus (HV); *n* = 29], bacteria (*Brucella* sp.; *n* = 7), protozoa (*Toxoplasma gondii*; *n* = 6), and helminths (*Nasitrema* sp.; *n* = 1). Aetiological diagnosis was established by molecular methods. Histopathologic evaluations of brain samples were performed in all the cases, and additional histochemical and/or immunohistochemical stains were carried out accordingly. Compared with those produced by other types of pathogens in our study, the characteristic features of viral meningoencephalitis (CeMV and HV) included the most severe and frequent presence of malacia, intranuclear, and/or intracytoplasmic inclusion bodies, neuronal necrosis and associated neuronophagia, syncytia and hemorrhages, predominantly in the cerebrum. The characteristic features of *Brucella* sp. meningoencephalitis included the most severe and frequent presence of meningitis, perivascular cuffing, cerebellitis, myelitis, polyradiculoneuritis, choroiditis, ventriculitis, vasculitis, and fibrinoid necrosis of vessels. The characteristic features of *T. gondii* meningoencephalitis included lymphocytic and granulomatous encephalitis, tissue cysts, microgliosis, and oedema. In the case of *Nasitrema* sp. infection, lesions are all that we describe since just one animal was available. The results of this study are expected to contribute, to a large extent, to a better understanding of brain-pathogen-associated lesions in cetaceans.

## Introduction

Infectious and inflammatory processes are among the most common causes of central nervous system involvement in stranded cetaceans (1–3). Meningoencephalitis of known infectious etiology can be caused by either bacteria, fungi, viruses, or protozoans (4).

Bacterial meningitis in cetaceans has been traditionally attributed to *Brucella* spp. *Brucella* spp. infection associated with meningitis, meningoencephalitis or meningoencephalomyelitis has been often reported in striped dolphins (*Stenella coeruleoalba*) (5–13), while only a few cases have been reported in the following cetacean species: Atlantic white-sided dolphin (*Lagenorhynchus acutus*) (14), short-beaked common dolphin (*Delphinus delphis*) (15), harbor porpoise (*Phocoena phocoena*) (16), long-finned pilot whale (*Globicephala melas*) (17), sperm whale (*Physeter macrocephalus*) (18), and common bottlenose dolphin (*Tursiops truncatus*) (19–21). Other bacterial pathogens have been more rarely reported in association with brain or meningeal abscess in stranded cetaceans: *Staphylococcus aureus* was isolated from a common bottlenose dolphin with acute pyogenic encephalitis and leptomeningitis (22); *Listeria monocytogenes* was identified, along with *Toxoplasma gondii* and *Brucella* spp., in a striped dolphin with severe meningoencephalitis (13); and *Vibrio parahaemolyticus* and *V. alginolyticus* were associated with meningoencephalitis in a bottlenose dolphin (23). Macro- and micro-abscessation of the central nervous system (CNS) and malacia are suggestive of bacterial infection. Mononuclear meningitis and choroiditis are also commonly found in neurobrucellosis (24, 25).

Fungal meningoencephalitis has been rarely described in cetaceans: it has been associated with *Cunninghamella bertholletiae* in a common bottlenose dolphin (26); with *Fusarium oxysporum* in a captive Atlantic bottlenose dolphin (27); with *Aspergillus fumigatus* in striped dolphins (2, 28), a northern bottlenose whale (*Hyperoodon ampullatus*) (29), and a harbor porpoise (*Phocoena phocoena*) (30); and with coccidioidomycosis in common bottlenose dolphins (31). Gross and microscopic haemorrhagic and necrotizing inflammations of the CNS are common features of fungal infection, as is a pyogranulomatous reaction associated with intralesional fungal structures. Vasculitis and thrombosis could also be present (24, 25).

Viral meningoencephalitis in cetaceans has been commonly associated with cetacean morbillivirus (CeMV) (32) and herpesvirus (HV) (33–36). Two cases have been associated with St. Louis encephalitis virus and West Nile virus in killer whales (37, 38). Classical CNS virus-associated lesions consist of meningeal mononuclear cell infiltrates, lymphoplasmacytic perivascular cuffs, microgliosis, intracytoplasmic and/or nuclear inclusion bodies (INCIBs), and neuronal necrosis and/or associated focal neuronophagia (24, 25).

Parasitic meningoencephalitis in cetaceans has been more frequently related to protozoans (*T. gondii*) (39–44). Reported lesions associated with toxoplasmosis in the CNS are non-suppurative meningoencephalitis and choroiditis, lymphoplasmacytic perivascular cuffs, gliosis, neuronal degeneration, and necrosis. Intra- or extracellular protozoan cysts have been occasionally observed, as have extracellular individual zoites. Trematodes of the genus *Nasitrema* sp. can also cause necrotizing and granulomatous encephalitis in cetaceans (45–48). A few cases of parasitic helminthic worms have also been reported to affect the CNS in cetaceans, most of which are related to nematodes of the genus *Crassicauda* (49, 50). CNS parasitic-associated lesions usually involve necrotizing encephalitis along the migratory path. Intralesional trematodes and nematodes (adults, eggs, and/or larvae) have been found (24, 25).

Despite the aforementioned CNS pathogen-associated lesions, meningitis, gliosis and perivascular cuffs are usually non-specific and may accompany various pathologies. In this paper, we systematically describe and compare the distinctive histopathological features of meningoencephalitis in stranded cetaceans induced by CeMV, HV, *Brucella* sp., *T. gondii* and *Nasitrema* sp.

## Materials and Methods

All the cases included in the present study were diagnosed during routine pathological and cause-of-death analyses in stranded cetaceans at the Division of Histology and Animal Pathology of the Institute for Animal Health (IUSA), Veterinary School, Universidad de Las Palmas de Gran Canaria. A prospective study on stranded cetaceans on the coasts of the Canarian Archipelago, and occasionally of other geographic regions, has been systematically carried out since 1999. The stranded animals were examined and necropsied according to standard procedures (51, 52). Stranding epidemiology (type, location and date) and life history data (species, age category, sex) were systematically recorded. During the necropsy, the body condition and the decomposition code of the carcass were also evaluated. Age categories were established based on total body length (53) and histologic gonadal examinations (54) in: neonate (animals with vibrissal hairs or vibrissal crypts, unhealed navel, fetal folds, and soft and folded dorsal fin and tail flukes), calf (animals with presence of milk in their stomach, or about the size of a nursing calf), juvenile (not sexually or physically mature animals), subadult (sexually but not physically mature animals), and adult (animals with mature gonads). Five codes of conservation condition were established (52): code 1 (extremely fresh carcass, just dead as an animal that has recently died or euthanized), code 2 (fresh carcass), code 3 (moderate decomposition), code 4 (advanced decomposition), and code 5 (mummified or skeletal remains). Four categories were established for body condition according to Joblon et al. (55): very poor (animals with extremely concave dorsal profile, visible costal reliefs, body fat low or absent, and fatty serous atrophy), poor (animals with concave dorsal profile, low body fat and the ribs can be noted by palpation, fair/moderate (animals with dorsal profile straight or slightly convex and moderate body fat), and good (animals with a dorsal convex profile and abundant body fat). During necropsy, formalin-fixed and fresh unfixed samples for histopathologic and virologic analyses, respectively, were prepared from selected tissues. CNS samples included cerebrum, cerebellum, brainstem and spinal cord. Before immersion in 4% formaldehyde solution in phosphate-buffered saline (PBS; pH 7.4) some longitudinal cuts (2–4) were made in both the cerebral and cerebellar hemispheres for a more rapid fixation of deep periventricular structures (56, 57). The fixed tissue samples were trimmed, routinely processed, embedded in paraffin, sectioned at a thickness of 5 μm, and stained with haematoxylin and eosin (HE) for examination by light microscopy. The unfixed samples were stored frozen at −80°C until processing for molecular virology testing.

### Histopathologic CNS Analysis

Thirty-eight animals with a morphological diagnosis of CNS inflammation and an associated aetiological diagnosis were included in the present study. Necropsy reports, including histopathological diagnostic reports of CNS, as well as epidemiologic and biologic data, photographic material, and ancillary diagnostic techniques, were retrieved and further analyzed. A few specimens included in this study have also been previously published.

Brain cortex lesions were systematically recorded (since they were consistently represented in the sample set), including subjective evaluations of meningitis, perivascular cuffing, microgliosis, malacia and neuronal necrosis and neuronophagia as absent (–), minimal (+), mild (++), moderate (+ + +), and severe (+ + ++), while INCIBs, hemorrhages and oedema were evaluated as absent (–) or present (+). Lesions affecting other regions and the presence of CNS-associated lesions were also described when present. An aetiological diagnosis of CNS inflammation was made based upon molecular techniques. However, when indicated by histopathological observations, histochemical [periodic acid Schiff (PAS) and Grocott] and/or immunohistochemical (IHC) [anti-*T. gondii*, anti-*Brucella* sp. and anti-canine distemper virus (CDV)] techniques were performed (2, 58). Appropriate positive and negative immunohistochemical controls (serial tissue sections in which primary antibodies were substituted by non-immune homologous serum) were included accordingly. Histological evidence of the involvement of other aetiological agents led to complementary analyses (microbiology).

### Molecular CNS Analysis

According to the histopathological diagnosis in each case, brain samples (*n* = 38) were screened for the presence of CeMV (*n* = 38; 100%), HV (*n* = 27; 71%), *Brucella* spp. (*n* = 24; 63.1%), *T. gondii* (*n* = 16; 42.1%), and *Nasitrema* spp. (*n* = 1; 2.6%). Approximately 0.5 g of fresh-frozen brain cortex (frontal lobe) from each animal was mechanically macerated in lysis buffer and subsequently centrifuged. DNA/RNA extraction was carried out from each 300 μL macerated sample by pressure filtration method, using a QuickGene® Mini 80 nucleid acid isolation instrument, using the DNA Tissue Kit S (QuickGene, Kurabo, Japan) according to the manufacturer's instructions with modifications: RNA carrier (Applied Biosystems^TM^, Thermo Fisher Scientific Waltham, Massachusetts, USA.) was added during the lysis step (59). Specifically, molecular detection of CeMV was performed by one or more of three different PCR methods: one-step RT-PCR of a 426-bp conserved region of the phosphoprotein (P) gene (31), RT-PCR using nested primers targeting the P gene (35), and one-step real-time RT-PCR to detect sequences in a conserved region (192 bp) of the fusion protein (F) gene (59). Herpesvirus DNA was detected by conventional nested PCR using degenerate primers designed to amplify a region of the DNA polymerase gene (60). *Brucella* spp. PCR assays were performed by two methods: quantitative duplex PCR amplifying a 150 bp fragment of the IS711 gene for the detection of *Brucella* at the genus level and the identification of genotype ST27 (61) or PCR using primers amplifying a 223-bp fragment of the bcsp31 gene (62, 63). *T. gondii* detection was carried out by two different assays: real-time PCR targeting a 529-bp repeat element of *T. gondii* (64) or newly developed real-time PCR. Specifically, primer sets (5′-CCTGGAAGGGCAGTGTTTAT-3′ and 5′-TGCCACGGTAGTCCAATACA-3′) were designed based on a 163 bp sequence within the *T. gondii* small subunit ribosomal RNA (18SrRNA) gene (GenBank accession No.: AY663792) using Primer3 software (http://bioinfo.ut.ee/primer3-0.4.0/primer3/). For *Nasitrema* spp. detection, primer sets (5′-CGGATTGGTTTTCGTTGTCT-3′ and 5′-ACCCAACCTAAGCAAGAGCA-3′) were generated and designed based upon the partial NADH dehydrogenase subunit 3 gene of *Nasitrema delphini*. (GenBank Accession no. KT180216), amplifying a fragment of approximately 230 bp, using Primer3 software (http://bioinfo.ut.ee/prime~r3-0.4.0/primer3/).

For both *T. gondii* and *Nasitrema* spp., amplification was performed in 20 μl of a reaction mixture containing 4 μl of template DNA, 1X SsoAdvanced™ Universal SYBR® Green Supermix (BioRad Laboratories, Hercules, CA), 0.375 μM of each primer and 5.250 μl of H_2_O treated with diethyl pyrocarbonate (DEPC). The real-time PCR cycle conditions were as follows: initial denaturation at 98°C for 3 min, template denaturation at 98°C for 15 s, followed by 40 amplification cycles of template denaturation at 98°C for 15 s, primer annealing at 60°C for 30 s and primer extension at 65°C for 5 s, with a final extension at 72°C for 5 s. The thermal cycler was a CFX96 Touch™ Real-Time PCR Detection System. The melting curve analysis was generated immediately after the amplification protocol by heating from 55 to 95°C in increments of 0.5°C/5 s. In order to establish the sensitivity and quantification dynamic range of these novel real-time PCR techniques, each positive control was diluted into ten-fold serial dilutions up to 10^−6^. The standard curve was measured in triplicate. Two negative controls (for extraction and amplification) and an amplification-positive control were included in each protocol.

The PCR products from positive cases were purified using a Real Clean spin kit (REAL) and sequenced (Sanger method). A BLAST search (www.ncbi.nlm.nih.gov/blast/Blast.cgi) was conducted to confirm the identity of the PCR amplicons.

## Results

We analyzed 38 animals with a histopathological diagnosis of meningitis, encephalitis or meningoencephalitis and an associated aetiological diagnosis, including 15 striped dolphins (39.5%), 10 Atlantic spotted dolphins (*Stenella frontalis*) (26.3%), five short-finned pilot whales (*Globicephala macrorhynchus*) (13.2%), three bottlenose dolphins (7.9%), three common dolphins (7.9%), one Cuvier's beaked whale (*Ziphius cavirostris*) (2.6%), and one Risso's dolphin (*Grampus griseus*) (2.6%). Epidemiologic and biologic data are summarized in Supplementary Table 1. Males represented a higher proportion (23/38; 60.5%) than females (15/38; 39.5%). Adults (*n* = 16) were overrepresented compared with juveniles (*n* = 8), subadults (*n* = 8), and calves (*n* = 6). More animals were found dead (*n* = 22; 57.9%) than visually confirmed to be live-stranded (*n* = 13; 34.2%) or found floating offshore (*n* = 2; 5.3%). Animals presented different body conditions: good (*n* = 10; 26.3%), moderate (*n* = 14; 36.8%), poor (*n* = 10; 26.3%), and very poor (*n* = 3; 7.9%). Neither stranding type nor body condition information was available for one animal (case no. 33). Twenty-five animals presented a “very fresh” (code 1) or “fresh” (code 2) post-mortem preservation status, 11 a “moderate post-mortem autolysis” condition (code 3) and two an “advanced post-mortem autolysis” condition (code 4). Animals were found stranded over a 17-years period (from March 2001 to July 2018) along the coast of the Canarian archipelago (*n* = 36) [Tenerife (*n* = 13), Gran Canaria (*n* = 9), Lanzarote (*n* = 8), and Fuerteventura (*n* = 6)] and Andalusia (*n* = 2).

Supplementary Table 2 summarizes the results of the molecular, immunohistochemical and histochemical assays. Sixteen animals tested positive for CeMV [eleven presenting the Dolphin Morbillivirus strain (DMV) and four the Pilot Whale Morbillivirus strain (PWMV)] by conventional and/or real-time RT-PCR and/or IHC. One animal was positive on immunohistochemistry for CDV but negative by PCR (35). All the PCR-positive animals were consistently immunostained against CDV antibody (2, 35). Thirteen animals tested positive for HV by conventional nested PCR. Seven animals tested positive for *Brucella* spp. by conventional and/or real-time PCR. Two of these seven *Brucella* sp.-PCR-positive animals were also tested by IHC, yielding positive results. Six animals tested positive for *T. gondii* by both IHC and real-time PCR. Only one animal was tested for *Nasitrema* spp. by real-time PCR, yielding positive results. Five animals presented co-infection: by CeMV and HV (3/5), by CeMV and *Brucella* sp. (1/5), and by HV and *Brucella* sp. (1/5). Histochemical stains (PAS and/or Grocott), used for a better visualization of suspected microscopic pathogens (tissue cysts and hyphae), were performed in seven animals. As a result, tissue cysts were seen in 4/6 histological sections of *T. gondii*-PCR-positive animals (cases 1, 3, 13, 16), and fungal hyphae (mucormycosis like) were identified in the brain of a CeMV-RT-PCR-positive animal (case 31). Microbiological tests revealed *Staphylococcus aureus* co-infection in case 34 (HV-PCR positive).

**Figure 1:**
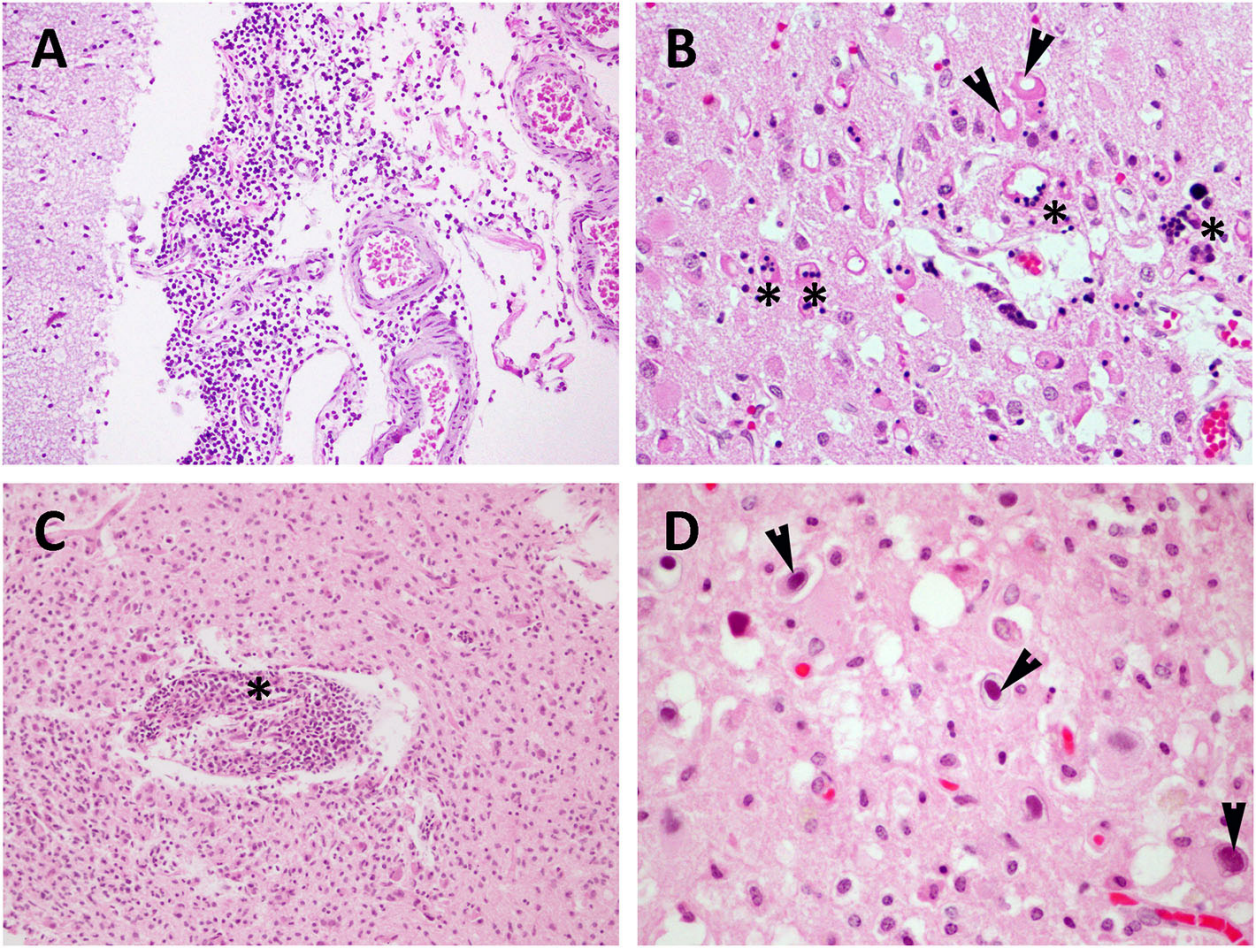
Viral (CeMV and HV) meningoencephalitis in stranded cetaceans. **(A)** CeMV-associated lesions in brain tissue samples from a DMV-positive Risso's dolphin (case 12). Moderate non-suppurative meningitis. Brain cortex. HE. 10×. **(B)** CeMV-associated lesions in brain tissue samples from a PWMV-positive short-finned pilot whale (case 31). Moderate presence of necrotic neurons (arrowheads) and syncytial cells (asterisks). Brain cortex. HE. 40×. **(C)** HV-associated lesions in brain tissue samples from an HV-positive Atlantic spotted dolphin (case 37). Perivascular cuffing (asterisk) and microgliosis. Brain cortex. HE. 20x. **(D)** HV-associated lesions in brain tissue samples from an HV-positive striped dolphin (case 29). Intranuclear inclusion bodies (arrowheads). Brain cortex. HE. 60× **(D)**.

CeMV was detected in 16 animals of five different species: striped dolphin (*n* = 8), short-finned pilot whale (*n* = 4), common dolphin (*n* = 2), bottlenose dolphin (*n* = 1), and Risso's dolphin (*n* = 1). Detailed CeMV-associated lesions in CNS are compiled in Table 1 and mainly consisted of minimal or mild non-suppurative meningitis (Figure 1A). It was moderate and severe in one animal, respectively. Animal presenting severe meningitis was also co-infected with *Brucella* sp. (case 6). Perivascular cuffing (consisting of lymphocytes and plasma cells) ranged from minimal to mild and moderate. Microgliosis was present in eleven animals, varying from minimal to mild. INCIBs were detected in two animals (cases 25 and 38). Malacia was only observed in four animals, ranging from minimal to mild. Neuronal necrosis and associated neuronophagia were detected in ten animals and ranged from minimal to mild to moderate (Figure 1B); the latter animal presented a mucormycosis-like fungal co-infection (case 31) and scattered polymorphonuclear neutrophils were observed within vessels and intermixed with the inflammatory infiltrate. Hemorrhages and oedema were present in nine and four animals, respectively. Lesions in other CNS regions included lymphoplasmacytic ventriculitis in one animal, cerebellitis in three animals, myelitis and choroiditis in four animals, respectively (cases 6, 9, 35, 38) and polyradiculoneuritis in one animal (case 6). Case 6 presented a CeMV and *Brucella* sp. co-infection, and cases 35 and 38 had CeMV and HV co-infection. Associated lesions included (pyo)granulomatous inflammation [specifically, granulomatous encephalitis (case 6), suppurative meningitis (case 9), and pyogranulomatous encephalitis (case 31, mucormycosis-like co-infection)], syncytia in four animals, vasculitis in three animals and neuronal degeneration and necrosis in one animal.

**Table 1:** CeMV-associated lesions in CNS: mild meningitis and perivascular cuffings, hemorrhages, and syncytia.

**Case no**.	**Cerebrum**								**Other regions**	**Associated lesions**	**Co-infections**	**References**
	**M**	**PC**	**Mg**	**INCIBs**	**Malacia**	**NN**	**H**	**O**				
4	+	++	+	–	–	–	–	+	Cerebellitis and myelitis	–	–	(35, 65)
6	++++	+++	+	–	+	–	+	–	Choroiditis. Ventriculitis and myelitis. Polyradiculoneuritis	Granulomatous encephalitis with multinucleated giant cells). Vasculitis	*Brucella* sp.	(21, 65, 66)
7	++	++	–	–	–	++	–	–	–	–	–	(65)
9	++	+	–	–	–	–	–	–	Choroiditis. Suppurative meningitis (Thalamus)	–	–	(35)
10	++	+++	+	–	–	+	+	+	Myelitis	–	HV	(35, 65)
11	+	+++	–	–	–	–	–	–	–	–	–	(35, 65)
12	+++	+++	+	–	+	+	–	–	–	–	–	(67)
14	++	++	+	–	–	+	+	+	–	–	–	(35, 65)
19	++	+++	+	–	–	+	+	–	Cerebellitis	–	–	(35, 65)
23	++	++	–	–	–	–	–	–	Cerebellitis and myelitis	–	–	–
24	+	++	–	–	–	–	+	+	Polioleucomielitis	–	–	–
25	++	++	+	+	–	++	+	–	–	Vasculitis. Syncytia	–	(65)
27	+	+++	+	–	–	+	+	–	–	–	–	–
31	+	+	++	–	++	+++	+	–	–	Pyogranulomatous encephalitis. Vasculitis. Syncytia	Mucormycosis like	(68)
35	+	+	+	–	–	+	–	–	Choroiditis	–	HV	
38	+	+	++	+	+	+	+	–	Choroiditis	Syncytia	HV	

*CeMV, Cetacean Morbillivirus; CNS, Central Nervous system; M, meningitis; PC, perivascular cuffing; Mg, microgliosis; INCIBs; intranuclear and/or intracytoplasmic inclusion bodies; NN, neuronal necrosis and associated focal neuronophagia; H, hemorrhages; O, oedema*.

HV infection was detected in 13 animals of five different species: striped dolphin (*n* = 7), Atlantic spotted dolphin (*n* = 3), bottlenose dolphin (*n* = 1), common dolphin (*n* = 1), and Cuvier's beaked whale (*n* = 1). HV-associated lesions are described in Table 2 and included non-suppurative meningitis in 10 animals and ranged from minimal to mild to severe (severe perivascular cuffings were observed in case 37). The latter was a case of HV and *Brucella* sp. co-infection. Perivascular cuffing (mainly composed of lymphocytes and plasma cells) was detected in 11 animals and ranged from minimal to mild, moderate (case 10) and severe (cases 29 and 37). Case 10 was a CeMV co-infection, and case 37 was a *Brucella* sp. co-infection. Microgliosis (Figure 1C) of different severity was observed in 10 animals: minimal and mild in six and four animals, respectively. INCIBs (Figure 1D) were present in six animals and were intranuclear in all of them. Malacia was minimal and mild in two and three animals, respectively. Neuronal necrosis and associated neuronophagia were detected in nine animals and ranged from minimal to mild. Hemorrhages and oedema were also present in six and two animals, respectively. Myelitis was present in two animals (cases 10 and 37). These two cases presented co-infections with CeMV and *Brucella* sp., respectively. Choroiditis was present in five animals (cases 29, 32, 34, 35, 38). Co-infection with CeMV was detected in cases 35 and 38 and with *S. aureus* in case 34. Syncytia were also present in a case co-infected with CeMV (case 38).

**Table 2:** HV-associated lesions in CNS, mild microgliosis, INIBs, mild malacia, and minimal neuronal necrosis and neuronophagia.

**Case no**.	**Cerebrum**								**Other regions**	**Associated lesions**	**Co-infections**	**References**
	**M**	**PC**	**Mg**	**INCIBs**	**Malacia**	**NN**	**H**	**O**				
2	–	+	+	–	–	–	–	+	–	–	–	(34)
10	++	+++	+	–	–	+	+	+	Myelitis	–	HV	(35, 65)
18	+	–	–	–	–	+	–	–	–	–	–	
21	++	++	+	+	++	+	+	–	–	–	–	
28	++	++	+	+	++	+	+	–	–	–	–	
29	++	++++	+	+	++	+	+	–	Choroiditis	–	–	
32	+	+	–	–	–	–	–	–	Choroiditis	–	–	
33	–	–	–	–	–	–	–	–	–	–	–	
34	–	++	++	–	–	–	+	–	Choroiditis	Suppurative meningoencephalitis	*S. aureus*	
35	+	+	+	–	–	+	–	–	Choroiditis	–	CeMV	
36	++	++	++	+	–	++	–	–	–	–	–	
37	++++	++++	++	+	+	++	–	–	Myelitis	–	*Brucella* sp.	
38	+	+	++	+	+	+	+	–	Choroiditis	Syncytia	CeMV	

*HV, Herpesvirus; CNS, Central Nervous System; M, meningitis; PC, perivascular cuffing; Mg, microgliosis; INCIBs; intranuclear and/or intracytoplasmic inclusion bodies; NN, neuronal necrosis and associated focal neuronophagia; H, hemorrhages; O, oedema*.

**Figure 2:**
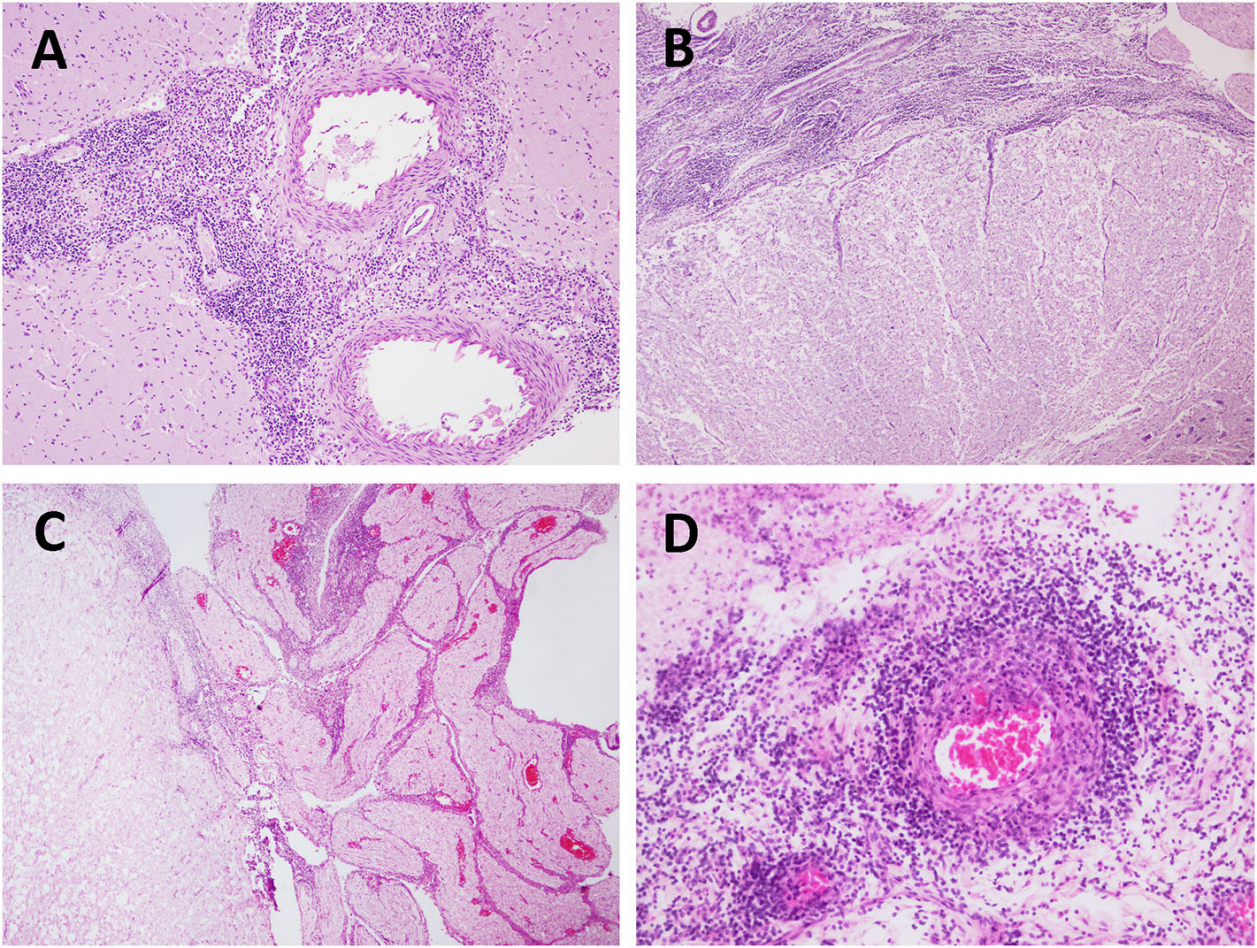
Bacterial (*Brucella* sp.) meningoencephalitis in stranded cetaceans. **(A)** Severe meningitis in a *Brucella* sp.-positive striped dolphin (case 30). Brain cortex. HE. 20×. **(B)** Severe meningomyelitis in a *Brucella*-sp.-positive striped dolphin (case 30). Spinal cord. HE 4×. **(C)** Meningomyelitis and polyradiculoneuritis in a *Brucella* sp.-positive striped dolphin (case 26). Spinal cord. HE 4×. HE. 10×. **(D)** Vasculitis in a *Brucella* sp.-positive striped dolphin (case 5). Spinal cord. HE. 20×.

*Brucella* spp. was detected in seven animals of five different species: striped dolphin (*n* = 2), Atlantic spotted dolphin (*n* = 2), bottlenose dolphin (*n* = 1), common dolphin (*n* = 1), and short-finned pilot whale (*n* = 1). The main *Brucella* spp.-associated lesions are compiled in Table 3 and included non-suppurative meningitis in all the animals, being severe in five animals, and, moderate in two animals (Figures 2A–C). Perivascular cuffings (mainly composed of lymphocytes and plasma cells) were detected in six animals and ranged from moderate in four animals to severe in two animals. Microgliosis was present in four animals, ranging from minimal to mild. Minimal malacia was present in two animals. Mild or moderate neuronal necrosis and associated neuronophagia were detected in one and two animals, respectively. Hemorrhages and/or oedema were not observed in this group of animals. Polyradiculoneuritis (Figure 2D) or neuritis (in five animals), ventriculitis (in one animal), choroiditis and cerebellitis (in three animals, respectively), and myelitis (in four animals) were the main lesions observed in other regions. Associated lesions included (pyo)granulomatous inflammation [specifically, pyogranulomatous meningitis (case 5), granulomatous encephalitis (case 6), and pyogranulomatous meningocerebellitis (case 22)], fibrinoid necrosis of vessels and vasculitis (Figure 2D) (in two animals, respectively).

**Table 3:** *Brucella* sp.-associated lesions in CNS: moderate to severe meningitis and perivascular cuffings, moderate neuronal necrosis and neuronophagia, moderate vasculitis and fibrinoid necrosis of vessels, ventriculitis, cerebellitis, myelitis, choroiditis, and polyradiculoneuritis.

**Case no**.	**Cerebrum**								**Other regions**	**Associated lesions**	**Co-infections**	**References**
	**M**	**PC**	**Mg**	**INCIBs**	**Malacia**	**NN**	**H**	**O**				
5	++++	–	–	–	–	–	–	–	Polyradiculoneuritis	Pyogranulomatous meningitis. Fibrinoid necrosis of vessels	–	–
6	++++	+++	+	–	+	–	+	–	Choroiditis. Ventriculitis and myelitis. Polyradiculoneuritis	Granulomatous encephalitis with multinucleated giant cells). Vasculitis	CeMV	(21, 65, 66)
8	++++	+++	–	–	–	–	–	–	Neuritis	–	–	–
22	+++	+++	–	–	–	–	–	–	Choroiditis. Pyogranulomatous meningocerebelitis	–	–	–
26	+++	+++	+	–	–	++	–	–	Myelitis. Cerebelitis. Polyneuritis	Fibrinoid necrosis of vessels. Intravascular bacteria	–	–
30	++++	++++	+	.	–	+	–	–	Choroiditis. Cerebelitis, myelitis and polyradiculoneuritis	Vasculitis	–	–
37	++++	++++	++	+	+	++	–	–	Myelitis	–	HV	

*CNS, Central Nervous System; M, meningitis; PC, perivascular cuffing; Mg, microgliosis; INCIBs; intranuclear and/or intracytoplasmic inclusion bodies; NN, neuronal necrosis and associated focal neuronophagia; H, hemorrhages; O, oedema*.

**Figure 3:**
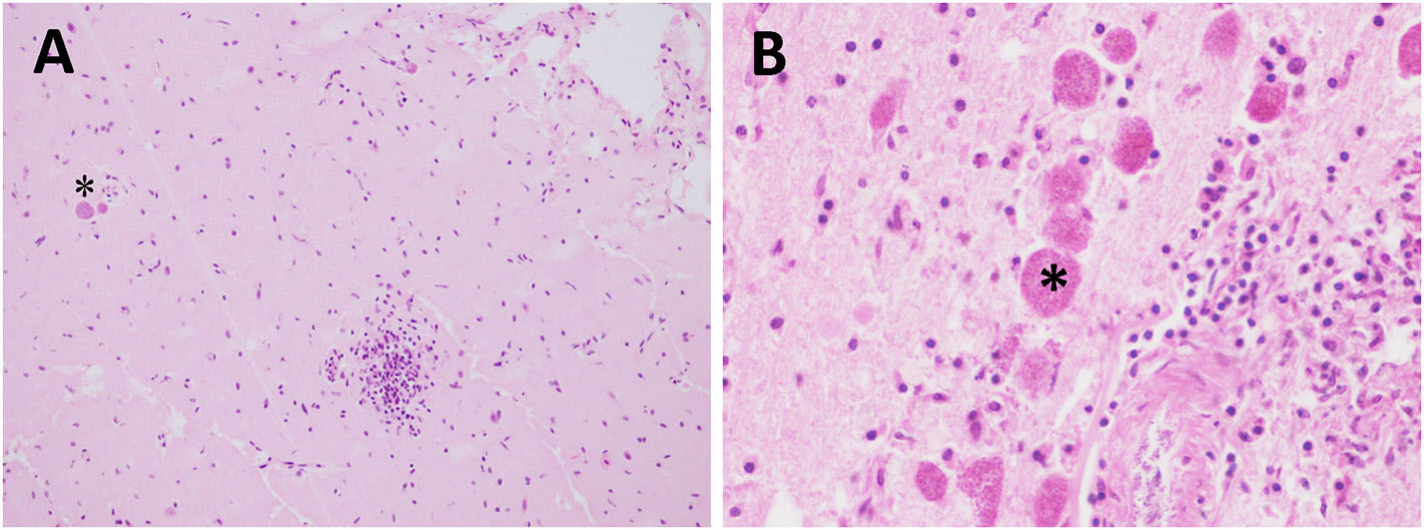
Protozoal (*T. gondii*) meningoencephalitis in stranded cetaceans. **(A)** Focal lymphohistiocytic to granulomatous inflammation in a *T. gondii*-positive Atlantic spotted dolphin. Two *T. gondii* protozoan cysts (asterisk) are seen within the same section (case 16). Cerebral cortex. HE. 40×. **(B)** Lymphohistiocytic to granulomatous inflammation with some associated *T. gondii* protozoan cysts (asterisk) (case 3). Cerebral cortex. HE. 60x.

All the animals testing positive for *T. gondii* were Atlantic spotted dolphins (*n* = 6). All *T. gondii*-associated lesions were of similar severity (detailed description in Table 4). Minimal meningitis and perivascular cuffing (Figure 3A) were present in five and six animals, respectively, and were largely composed of lymphocytes with few plasma cells or macrophages. Minimal microgliosis was observed in four animals. Minimal or mild malacia was observed in two and one animals, respectively. Mild or minimal neuronal necrosis and neuronophagia were detected in one and three animals, respectively. Hemorrhages were detected in one animal and oedema in two animals. Other lesions detected in this group of animals included choroiditis and cerebellitis in one and two animals, respectively. Associated lesions included granulomatous inflammation (specifically granulomatous encephalitis) (Figure 3B) in all the animals and the presence of tissue cysts (confirmed by PAS staining and IHC) in four animals.

**Table 4:** *T.gondii*-associated lesions in CNS: minimal meningitis and perivascular cuffings, minimal microgliosis, minimal malacia, mild neuronal necrosis and neuronophagia, oedema, pyogranulomatous inflammation, and tissue cysts.

**Case no**.	**Cerebrum**								**Other regions**	**Associated lesions**	**Co-infections**	**References**
	**M**	**PC**	**Mg**	**INCIBs**	**Malacia**	**NN**	**H**	**O**				
1	–	+	+	–	+	++	–	+	Choroiditis. Granulomatous cerebellitis	Lymphocytic to granulomatous encephalitis. Tissue cysts	–	–
3	+	+	+	–	+	+	–	–	Granulomatous cerebellitis	Lymphocytic to granulomatous encephalitis. Tissue cysts	–	–
13	+	+	–	–	–	–	–	–	–	Lymphocytic to granulomatous encephalitis. Tissue cysts	–	–
15	–	+	+	–	–	+	+	+	–	Lymphocytic to granulomatous encephalitis	–	–
16	+	+	+	–	++	+	–	–	–	Lymphocytic to granulomatous encephalitis. Tissue cysts	–	–
17	+	+	–	–	–	–	–	+	–	Lymphocytic to granulomatous encephalitis	–	–

*T. gondii, Toxoplasma gondii; M, meningitis; PC, perivascular cuffing; Mg, microgliosis; INCIBs; intranuclear and/or intracytoplasmic inclusion bodies; NN, neuronal necrosis and associated focal neuronophagia; H, hemorrhages; O, oedema*.

*Nasitrema* sp. was detected in a bottlenose dolphin (case 20). *Nasitrema* sp.-associated lesions included minimal non-suppurative meningitis and perivascular cuffing and moderate malacia. Oedema was also present. Lesions in other regions included meningomyelitis. Associated lesions were pyogranulomatous inflammation, vascular necrosis and vasculitis, and intralesional sections of characteristic golden-brown triangular eggs.

Number and percentages of animals displaying every type of lesion (including the severity of morphological lesion) grouped by associated pathogens are available in Table 5.

**Table 5:** Percentages and number of animals presenting each lesion grouped by etiologies. Higher number and percentages are underlined.

**Brain-associated lesions**	**CeMV (*****n*** **=** **16)**		**HV (*****n*** **=** **13)**		***Brucella*** **sp. (*****n*** **=** **7)**		***T. gondii*** **(*****n*** **=** **6)**		***Nasitrema*** **sp. (*****n*** **=** **1)**	
	**Animals (*n*)**	**Percentage (%)**	**Animals (*n*)**	**Percentage (%)**	**Animals (*n*)**	**Percentage (%)**	**Animals (*n*)**	**Percentage (%)**	**Animals (*n*)**	**Percentage (%)**
**Meningitis**	**16**	**100**	**10**	**76.9**	**7**	**100**	**5**	**83.3**	**1**	**100**
Minimal	7	43.7	4	30.8	0	0	5	83.3	1	100
Mild	7	43.7	5	38.5	0	0	0	0	0	0
Moderate	1	6.25	0	0	2	28.6	0	0	0	0
Severe	1	6,25	1	7.7	5	71.4	0	0	0	0
**Perivascular cuffings**	**16**	**100**	**11**	**84.6**	**6**	**85.7**	**6**	**100**	1	100
Minimal	4	25	4	30.8	0	0	6	100	1	100
Mild	6	37.5	4	30.8	0	0	0	0	0	0
Moderate	6	37.5	1	7.7	4	57.1	0	0	0	0
Severe	0	0	2	15.4	2	28.6	0	0	0	0
**Microgliosis**	**11**	**68.7**	**10**	**76.9**	**4**	**57.1**	**4**	**66.7**	0	0
Minimal	9	56.22	6	46.1	3	42.9	4	66.7	0	0
Mild	2	12.5	4	30.8	1	14.3	0	0	0	0
Moderate	0	0	0	0	0	0	0	0	0	0
Severe	0	0	0	0	0	0	0	0	0	0
**INCIBs**	**2**	**12.5**	**6**	**46.1**	**0**	**0**	**0**	**0**	0	0
**Malacia**	**4**	**25**	**5**	**38.5**	**2**	**28.6**	**3**	**50**	**1**	**100**
Minimal	3	18.7	2	12.5	2	28.6	2	33.3	0	0
Mild	1	6.2	3	23.1	0	0	1	16.7	0	0
Moderate	0	0	0	0	0	0	0	0	1	100
Severe	0	0	0	0	0	0	0	0	0	0
**Neuronal necrosis and neuronophagia**	**10**	**62.5**	**9**	**69.2**	**3**	**42.9**	**4**	**66.7**	**0**	**0**
Minimal	7	43.7	7	53.8	0	0	3	50	0	0
Mild	2	12.5	2	15.4	1	14.3	1	16.7	0	0
Moderate	1	6.25	0	0	2	28.6	0	0	0	0
Severe	0	0	0	0	0	0	0	0	0	0
**Hemorrhages**	**9**	**56.2**	**6**	**46.1**	**0**	**0**	**1**	**16.7**	**0**	**0**
**Oedema**	**4**	**25**	**2**	**15.4**	**0**	**0**	**2**	**33.3**	**0**	**0**
**Associated brain lesions**	**11**	**68.75**	**1**	**7.7**	**4**	**57.1**	**6**	**100**	**0**	**0**
Pyogranulomatous inflammation	3	18.7	0	0	3	42.8	6	100	0	0
Syncytia	4	25	1	7.7	0	0	0	0	0	0
Vasculitis	3	18.7	0	0	2	28.6	0	0	0	0
Fibrinoid necrosis of vessels	0	0	0	0	2	28.6	0	0	0	0
Tissue cysts	0	0	0	0	0	0	4	66.7	0	0
**Lesions in other regions**	**13**	**81.25**	**7**	**53.8**	**7**	**100**	**2**	**33.3**	0	0
Ventriculitis	1	6.2	0	0	1	14.3	0	0	0	0
Cerebellitis	3	16	0	0	3	42.9	2	33.3	0	0
Myelitis	4	25	2	15.4	4	57.1	0	0	1	100
choroiditis	4	25	5	38.5	3	42.9	1	16.7	0	0
Polyradiculoneuritis	1	6.2	0	0	5	71.4	0	0	0	0

*CeMV, Cetacean Morbillivirus; HV, Herpesvirus; T. gondii, Toxoplasma gondii. Bold numbers indicate n and percentages of main CNS lesions observed in each group of etiological agents*.

## Discussion

Meningitis and encephalitis are among the leading known natural causes of death in stranded cetaceans and may be caused by a wide range of pathogens. In some cases, brain is the only organ affected, thus, many infectious diseases may be overlooked if brain is not carefully investigated. This study describes histopathological findings in post-mortem brain tissue specimens from stranded cetaceans associated with five relevant infectious agents: viruses (CeMV and HV; *n* = 29), bacteria (*Brucella* sp.; *n* = 7), protozoan (*T. gondii*; *n* = 6) and helminths (*Nasitrema* sp.; *n* = 1). Aetiological diagnosis was established by molecular methods. Characteristic, but not pathognomonic, histological alterations associated with each infection are discussed below and compared. In the case of *Nasitrema* sp. infestation, lesions are just described since just one animal was available.

As in humans, viral meningoencephalitis is the most common type of meningoencephalitis in cetaceans. Most of the animals from our study (76.3%) presented viral meningoencephalitis. Sixteen animals with a molecular diagnosis of CeMV infection in the CNS, representing 42.1% of the total animals screened for this pathogen in our study, and 13 animals with a molecular diagnosis of HV infection in the CNS, representing 48.1% of the total animals screened for this pathogen in our study, are reported.

CeMVs are RNA viruses responsible for massive die-offs worldwide and include three well-characterized strains (porpoise morbillivirus, DMV, and PWMV) and three less well-characterized strains detected in Hawaii and in the southern hemisphere (32). CeMV is a well-recognized neurotropic pathogen and localized brain lesions have been described in cetaceans that have cleared systemic CeMV infection, resembling subacute sclerosing panencephalitis (SSPE) and old dog encephalitis (ODE) (20, 32, 65). IHC labeling has been successfully used to diagnose CeMV preferentially in earlier stages of the disease. Herpes simplex virus encephalitis is the most common cause of sporadic fatal encephalitis in humans worldwide. In cetaceans, alphaherpesvirus (*Herpesviridae* family) infections have been less described than gammaherpesvirus infections, and associated lesions range from incidental and asymptomatic to necrotizing inflammation in different organ systems. Classical CNS lesions have been associated with the presence of alphaherpesviruses in some cases (33–36). Immunohistochemistry has failed to consistently highlight HV-infected cells in brain samples in cetaceans (immunoreactivity has been proven in only two studies) (33, 69).

In our study, CeMV- and HV-associated brain lesions were in concordance with previous descriptions (20, 32). Comparing these results to those for lesions caused by other aetiological agents under study, we observed that mild malacia was more frequently detected in HV-positive animals, while it was mainly minimal in animals infected by *T. gondii, Brucella* sp., and CeMV. INCIBs were only detected in animals positive for HV or CeMV, being intranuclear in HV-infected animals. Neuronal necrosis and associated neuronophagia were predominantly minimal in animals positive for HV, *T. gondii*, and CeMV. Syncytia were more frequently detected in CeMV-positive animals. In one HV-positive animal, the presence of syncytia was reported (case 38, also co-infected by CeMV). Hemorrhages were present, with decreasing frequency in animals positive for CeMV, HV, and *T. gondii*. These features and the neuroanatomical distribution of lesions in CeMV-positive animals from our study mostly fit the chronic stage of the disease and localized brain lesions (“brain-only form of DMV infection”), although the presence of neuronal necrosis, ICNIBs and syncytia, detected in three CeMV-positive cases, are more related to the acute and subacute stages of the infection (32, 65). The differences in features between CeMV and HV are the presence of eosinophilic, basophilic or amphophilic intranuclear inclusions in neurons and glial cells in HV-positive animals and eosinophilic intranuclear and intracytoplasmic inclusions in the same types of cells in CeMV-positive animals. The presence of syncytia was attributable to CeMV.

Bacterial meningoencephalitis was the second most common cause of encephalitis in our study (18.4%). Seven animals with a molecular diagnosis of *Brucella* sp. infection in the CNS were found, representing 29.2% of the total animals screened for this pathogen in our study. Two *Brucella* sp.-PCR-positive animals were immunostained with the *Brucella* antibody (21, 58). Brucella infection is reported for the first time in the short-finned pilot whale species (*n* = 1). The information presented here increases the number of confirmed *Brucella* sp.-positive cases within the Canarian archipelago from two previously reported cases to seven.

Brucellosis is a worldwide zoonosis characterized by its clinical polymorphism. In humans, neurobrucellosis (NB) is an uncommon complication of the infection (occurring in 0.5–25% of cases), in which meningeal involvement is the most common presentation (70–72). Human NB also includes encephalitis, myelitis, radiculoneuritis, brain or epidural abscesses, granuloma, and demyelinating and meningovascular syndromes (73). In cetacean brucellosis, the most frequent lesions involve the CNS; the cerebellum, brainstem, spinal cord, and medulla oblongata, with less frequent involvement of the cerebral cortex, are the most consistently affected regions (8, 20). *Brucella* species antigens in phagocytic cells can be highlighted by IHC (20, 58). In our study, histological analysis revealed *Brucella* sp.-associated brain lesions of different severity and frequency matching previous descriptions (8, 20). Comparing these results to those for lesions caused by other aetiological agents under study, we observed the severest lesions in the meninges of animals with a molecular diagnosis of *Brucella* sp. infection; the majority of *Brucella* sp.-PCR-positive animals displayed severe non-suppurative meningitis, while most of the animals positive for CeMV and HV displayed mild non-suppurative meningitis, compared with minimal presentation of the lesion in the majority of *T. gondii*-positive animals. Encephalitis with lymphoplasmacytic perivascular cuffing was also more pronounced among the *Brucella* sp.-positive animals in our study; it was moderate in most of *Brucella* sp.-positive animals, mild in most of CeMV-positive animals, mild and minimal in the same proportion in HV-positive animals, and minimal in all of *T. gondii*-positive animals. Cerebellitis was observed mainly in *Brucella* sp.-positive animals and less frequently in animals positive for CeMV or *T. gondii*. Myelitis was more frequently detected within *Brucella* sp.-PCR-positive animals than in animals infected by other pathogens, such as CeMV and HV. Polyradiculoneuritis was only observed in CNS samples of animals infected by two different pathogens, with a higher prevalence in *Brucella* sp.-positive animals than CeMV-positive animals (case 6 was also co-infected by *Brucella* sp.). A predisposition to cranial nerve involvement in NB could be due to the pathogen's predilection for the base of the cranium (74). Choroiditis was a more common finding in animals from our study infected by *Brucella* sp. than in those infected by other pathogens, such as HV, CeMV, and *T. gondii*. Lymphoplasmacytic ventriculitis was more common in *Brucella* sp.-infected animals than in CeMV-positive animals. Vasculitis and fibrinoid necrosis of vessels were also more common among *Brucella* sp.-infected animals from our study than in animals infected with other pathogens. Vasculitis was also observed in a low percentage in CeMV-positive animals.

Protozoan meningoencephalitis was the third most common cause of encephalitis in our study (15.8%). Six animals with a molecular diagnosis of *T. gondii* infection in the CNS were seen, representing 37.5% of the total animals screened for this pathogen in our study. All the PCR-positive animals consistently immunostained for *T. gondii* antibody (1, 2).

*Toxoplasma gondii* is a neurotropic protozoan globally distributed among mammalian hosts, including humans. Non-suppurative meningoencephalitis due to *T. gondii* has been sporadically described in cetaceans (1, 2, 20, 43). Tissue cysts and zoites are confirmed by IHC labeling (2, 43). In our study, histological analysis evidenced *T. gondii*-associated brain lesions of different severity and frequency. These lesions are similar, except for the less prominent perivascular cuffing, to previous descriptions (20, 43). Comparing these results to those for lesions caused by other aetiological agents under study, we observed that minimal microgliosis was a common feature caused by *T. gondii* and by the different pathogens under study: CeMV, HV, and *Brucella* sp. Oedema was more frequently present in animals positive for *T. gondii* than in animals positive for CeMV or HV. Granulomatous (in addition to lymphocytic) encephalitis was observed in all *T. gondii*-positive animals, higher than the rates in animals positive for CeMV and *Brucella* sp. Tissue cysts of *T. gondii* were frequently observed.

Helminthic meningoencephalitis was underrepresented in our study (2.6%), since just one animal was tested for *Nasitrema* sp., yielding positivity.

*Nasitrema* spp. are trematodes that normally inhabit the pterygoid sinuses and tympanic cavities of odontocetes (75). Non-suppurative meningoencephalitis has been associated with aberrant migration of this parasite. Sections of adult trematodes and eggs surrounded by multinucleated giant cells can be detected within parenchymal brain lesions. Neuritis of the eighth cranial nerve and otitis media can be occasionally present (20). Histological features of *Nasitrema* sp.-positive animal from our study included minimal meningoencephalitis, mild malacia, oedema, myelitis, pyogranulomatous encephalitis, vasculitis and fibrinoid necrosis of vessels.

In summary, compared with those produced by other pathogens in our study, the characteristic features of viral meningoencephalitis (CeMV and HV) included the most severe and frequent presence of malacia, INCIBs, neuronal necrosis and associated neuronophagia, syncytia and hemorrhages, predominantly in the cerebrum. The characteristic features of *Brucella* sp. meningoencephalitis includes the most severe and frequent presence of meningitis, perivascular cuffing, cerebellitis, myelitis, polyradiculoneuritis, choroiditis, ventriculitis, vasculitis, and fibrinoid necrosis of vessels. The characteristic features of *T. gondii* meningoencephalitis include lymphocytic and granulomatous encephalitis, tissue cysts, microgliosis, and oedema. However, histopathological findings in these cases can be influenced by superimposition by simultaneous or secondary infections. Specifically, three animals presented CeMV and HV brain co-infection; one case was co-infected by CeMV and *Brucella* sp.; and one was co-infected by HV and *Brucella* sp. Co-infection by HV and *S. aureus* and co-infection by CeMV and mucormycosis-like lesions were detected in one animal, respectively.

## Conclusion

A multidisciplinary approach is needed for the early detection and surveillance of emerging and reemerging pathogens (76). Histopathological findings may suggest a list of differential diagnoses, but the use of additional laboratory techniques (microbiology, IHC and/or PCR) is essential to determine the specific infectious etiology. However, when these methods (specially molecular assays) are not accessible or fail to identify causative agents, histopathology is particularly useful as part of this multidisciplinary approach. The results of this study are expected to contribute, to a large extent, to a better understanding of brain-pathogen-associated lesions in cetaceans.

## Data Availability Statement

The datasets generated for this study are available on request to the corresponding author.

## Ethics Statement

Ethical review and approval was not required for the animal study because no experiments were performed on live animals because our work was based on dead stranded cetaceans, and the field studies did not involve endangered or protected species.

## Author Contributions

ES analyzed the data, drafted the manuscript, contributed to the gross, histological and molecular diagnosis of the cases, and designed new molecular assays. AF contributed to the gross and histological diagnosis of the cases and guided the ES during the drafting and publication process. IF-J contributed to the molecular analysis of the cases. DZ contributed to the histopathological and immunohistochemical diagnosis of the cases. JD-D contributed to the gross and histological diagnosis of the cases. RP-L, NC, FC, PD-S, and CS-S contributed to the gross and histological diagnosis of the cases. MA contributed to the gross diagnosis of the cases and guided the ES during the drafting process. All authors gave final approval of the version to be published.

## Conflict of Interest

The authors declare that the research was conducted in the absence of any commercial or financial relationships that could be construed as a potential conflict of interest.

## References

[1] Arbelo M Los Monteros AE Herraez P Andrada M Sierra E Rodriguez F. Pathology and causes of death of stranded cetaceans in the Canary Islands (1999-2005). Dis Aquat Org. (2013) 103:87–99. 10.3354/dao02558 23548359

[2] Diaz-Delgado J Fernandez A Sierra E Sacchini S Andrada M Vela AI. Pathologic findings and causes of death of stranded cetaceans in the Canary Islands (2006-2012). PLoS ONE. (2018) 13:e0204444. 10.1371/journal.pone.0204444 30289951 PMC6173391

[3] Pintore MD Mignone W Di Guardo G Mazzariol S Ballardini M Florio CL Neuropathologic findings in cetaceans stranded in Italy (2002–2014). J Wildl Dis. (2018) 54:295–303. 10.7589/2017-02-035 29369721

[4] Dash SK Herpes meningoencephalitis: causes, diagnosis, and treatment. In: Pana M editor. Meningoencephalitis: Disease Which Requires Optimal Approach in Emergency Manner. London, UK: IntechOpen (2017). p. 49–60. 10.5772/intechopen.68553

[5] Foster G Macmillan AP Godfroid J Howie F Ross HM Cloeckaert A. A review of Brucella sp. infection of sea mammals with particular emphasis on isolates from Scotland. Vet Microbiol. (2002) 90:563–80. 10.1016/S0378-1135(02)00236-5 12414172

[6] Gonzalez L Patterson IA Reid RJ Foster G Barberan M Blasco JM. Chronic meningoencephalitis associated with Brucella sp. infection in live-stranded striped dolphins (*Stenella coeruleoalba*). J Comp Pathol. (2002) 126:147–52. 10.1053/jcpa.2001.0535 11945003

[7] Muñoz PM Garcia-Castrillo C Lopez-Garcia P Gonzalez-Cueli JC De Miguel MJ Marin CM. Isolation of *Brucella* species from a live-stranded striped dolphin (*Stenella coeruleoalba*) in Spain. Vet Rec. (2006) 158:450–1. 10.1136/vr.158.13.450 16581998

[8] Hernandez-Mora G Gonzalez-Barrientos R Morales JA Chaves-Olarte E Guzman-Verri C Barquero-Calvo E. Neurobrucellosis in stranded dolphins, Costa Rica. Emerging Infect Dis. (2008) 14:1430–3. 10.3201/eid1409.071056 18760012 PMC2603106

[9] Davison NJ Cranwell MP Perrett LL Dawson CE Deaville R Stubberfield EJ. Meningoencephalitis associated with Brucella species in a live-stranded striped dolphin (*Stenella coeruleoalba*) in south-west England. Vet Rec. (2009) 165:86–9. 10.1136/vetrec.165.3.86 19617615

[10] Gonzalez-Barrientos R Morales JA Hernandez-Mora G Barquero-Calvo E Guzman-Verri C Chaves-Olarte E. Pathology of striped dolphins (*Stenella coeruleoalba*) infected with Brucella ceti. J Comp Pathol. (2010) 142:347–52. 10.1016/j.jcpa.2009.10.017 19954790

[11] Alba P Terracciano G Franco A Lorenzetti S Cocumelli C Fichi G. The presence of *Brucella ceti* ST26 in a striped dolphin (*Stenella coeruleoalba*) with meningoencephalitis from the Mediterranean Sea. Vet Microbiol. (2013) 164:158–63. 10.1016/j.vetmic.2013.01.023 23419820

[12] Isidoro-Ayza M Ruiz-Villalobos N Perez L Guzman-Verri C Munoz PM Alegre F. *Brucella ceti* infection in dolphins from the Western Mediterranean sea. BMC Vet Res. (2014) 10:206. 10.1186/s12917-014-0206-7 25224818 PMC4180538

[13] Grattarola C Giorda F Iulini B Pintore MD Pautasso A Zoppi S. Meningoencephalitis and Listeria monocytogenes, Toxoplasma gondii and Brucella spp. coinfection in a dolphin in Italy. Dis Aqu Organ. (2016) 118:169–74. 10.3354/dao02957 26912047

[14] Dagleish MP Barley J Howie FE Reid RJ Herman J Foster G. Isolation of *Brucella* species from a diseased atlanto-occipital joint of an Atlantic white-sided dolphin (*Lagenorhynchus acutus*). Vet Rec. (2007) 160:876–8. 10.1136/vr.160.25.876 17586794

[15] Davison NJ Barnett JE Perrett LL Dawson CE Perkins MW Deaville RC. Meningoencephalitis and arthritis associated with Brucella ceti in a short-beaked common dolphin (*Delphinus delphis*). J Wildl Dis. (2013) 49:632–6. 10.7589/2012-06-165 23778612

[16] Jauniaux TP Brenez C Fretin D Godfroid J Haelters J Jacques T. *Brucella ceti* infection in harbor porpoise (*Phocoena phocoena*). Emerg Infect Dis. (2010) 16:1966–8. 10.3201/eid1612.101008 21122233 PMC3294555

[17] Davison NJ Brownlow A Mcgovern B Dagleish MP Perrett LL Dale EJ. First report of Brucella ceti-associated meningoencephalitis in a long-finned pilot whale Globicephala melas. Dis Aquat Org. (2015) 116:237–41. 10.3354/dao02926 26503778

[18] West KL Levine G Jacob J Jensen B Sanchez S Colegrove K. Coinfection and vertical transmission of Brucella and Morbillivirus in a neonatal sperm whale (Physeter macrocephalus) in Hawaii, USA. J Wildl Dis. (2015) 51:227–32. 10.7589/2014-04-092 25390763

[19] Venn-Watson S Colegrove KM Litz J Kinsel M Terio K Saliki J. Adrenal gland and lung lesions in gulf of mexico common bottlenose dolphins (*Tursiops truncatus*) found dead following the deepwater horizon oil spill. PLoS ONE. (2015) 10:e0126538. 10.1371/journal.pone.0126538 25992681 PMC4439104

[20] St Leger JASRAM Cetacea. In: Terio KA St Leger J. Editors. Pathology of Wildlife Zoo Animals. Cambridge, MA: Academic Press (2018). p. 553–68. 10.1016/B978-0-12-805306-5.00022-5

[21] Sierra E Fernandez A Felipe-Jimenez I Zucca D Di Francesco G Diaz-Delgado J. Neurobrucellosis in a common bottlenose dolphin (*Tursiops truncatus*) stranded in the Canary Islands. BMC Vet Res. (2019) 15:353. 10.1186/s12917-019-2089-0 31638986 PMC6805616

[22] Colgrove GS Migaki G. Cerebral abscess associated with stranding in a dolphin. J Wildl Dis. (1976) 12:271–4. 10.7589/0090-3558-12.2.271 933321

[23] Di Renzo L Di Francesco G Profico C Di Francesco CE Ferri N Averaimo D. Vibrio parahaemolyticus- and V. alginolyticus-associated meningo-encephalitis in a bottlenose dolphin (*Tursiops truncatus*) from the Adriatic coast of Italy. Res Vet Sci. (2017) 115:363–5. 10.1016/j.rvsc.2017.06.023 28709108

[24] Summers BA Cummings JF Delahunta A Veterinary Neuropathology. St Louis, MO: Mosby (1995).

[25] Vandevelde M Higgins R Oevermann A Veterinary Neuropathology: Essentials of Theory and Practice. Wiley (2012).

[26] Isidoro-Ayza M Perez L Cabanes FJ Castella G Andres M Vidal E. Central nervous system mucormycosis caused by Cunninghamella bertholletiae in a bottlenose dolphin (*Tursiops truncatus*). Gangoso L. (2014) 50:634–8. 10.7589/2013-10-284 24807173

[27] Staggs L St Leger J Bossart G Townsend FI Jr Hicks C Rinaldi M. A novel case of Fusarium oxysporum infection in an Atlantic bottlenose dolphin (*Tursiops truncatus*). J Zoo Wildl Med. (2010) 41:287–90. 10.1638/2009-0037R2.1 20597220

[28] Domingo M Visa J Pumarola M Marco AJ Ferrer L Rabanal R. Pathologic and immunocytochemical studies of morbillivirus infection in striped dolphins (*Stenella coeruleoalba*). Vet Pathol. (1992) 29:1–10. 10.1177/030098589202900101 1557861

[29] Dagleish MP Foster G Howie FE Reid RJ Barley J. Fatal mycotic encephalitis caused by Aspergillus fumigatus in a northern bottlenose whale (*Hyperoodon ampullatus*). Davison, N J. (2008) 163:602–4. 10.1136/vr.163.20.602 19011249

[30] Dagleish MP Patterson IA Foster G Reid RJ Linton C Buxton D. Intracranial granuloma caused by asporogenic Aspergillus fumigatus in a harbour porpoise (Phocoena phocoena). Vet Rac. (2006) 159:458–60. 10.1136/vr.159.14.458 17012612

[31] Reidarson TH Mcbain JF Dalton LM Rinaldi MG “Mycotic diseases. In: Fmd DLY Raton GB editors. CRC Handbook of Marine Mammal Medicine: Health, Disease Rehabilitation. Florida, FA: CRC Press (2001). 10.1201/9781420041637.ch17

[32] Van Bressem MF Duignan PJ Banyard A Barbieri M Colegrove KM De Guise S. Cetacean morbillivirus: current knowledge and future directions. Viruses. (2014) 6:5145–81. 10.3390/v6125145 25533660 PMC4276946

[33] Kennedy S Lindstedt IJ Mcaliskey MM Mcconnell SA Mccullough SJ Herpesviral encephalitis in a harbor porpoise (*Phocoena phocoena*). J Zoo Wildl Med 23:374–9.

[34] Esperón F Fernandez A Sanchez-Vizcaino JM. Herpes simplex-like infection in a bottlenose dolphin stranded in the Canary Islands. Dis Aquat Org. (2008) 81:73–6. 10.3354/dao01915 18828564

[35] Sierra E Sanchez S Saliki JT Blas-Machado U Arbelo M Zucca D. Retrospective study of etiologic agents associated with nonsuppurative meningoencephalitis in stranded cetaceans in the canary islands. J Clin Microb. (2014) 52:2390–7. 10.1128/JCM.02906-13 24759718 PMC4097689

[36] Van Elk C Van De Bildt M Van Run P De Jong A Getu S Verjans G. Central nervous system disease and genital disease in harbor porpoises (Phocoena phocoena) are associated with different herpesviruses. Vet Res. (2016) 47:016–0310. 10.1186/s13567-016-0310-8 26861818 PMC4748569

[37] Buck C Paulino GP Medina DJ Hsiung GD Campbell TW Walsh MT. Isolation of St. Louis encephalitis virus from a killer whale. Clin Diagn Virol. (1993) 1:109–12. 10.1016/0928-0197(93)90018-Z 15566723

[38] St Leger J Wu G Anderson M Dalton L Nilson E Wang D. West Nile virus infection in killer whale, Texas, USA, 2007. Emerging Infect Dis. (2011) 17:1531–3. 10.3201/eid1708.101979 21801643 PMC3381582

[39] Mikaelian I Boisclair J Dubey JP Kennedy S Martineau D. Toxoplasmosis in beluga whales (Delphinapterus leucas) from the St Lawrence estuary: two case reports and a serological survey. J Comp Pathol. (2000) 122:73–6. 10.1053/jcpa.1999.0341 10627393

[40] Jardine JE Dubey JP. Congenital toxoplasmosis in a Indo-Pacific bottlenose dolphin (Tursiops aduncus). J Parasitol. (2002) 88:197–9. 10.1645/0022-3395(2002)088[0197:CTIAIP]2.0.CO;2 12053968

[41] Resendes AR Almería S Dubey JP Obón E Juan-Sallés C Degollada E. Disseminated toxoplasmosis in a mediterranean pregnant risso's dolphin (*Grampus griseus*) with transplacental fetal infection. J Parasitol. (2002) 88:1029–32. 10.1645/0022-3395(2002)088[1029:DTIAMP]2.0.CO;2 12435153

[42] Dubey JP Morales JA Sundar N Velmurugan GV Gonzalez-Barrientos CR Hernandez-Mora G. Isolation and genetic characterization of Toxoplasma gondii from striped dolphin (*Stenella coeruleoalba*) from Costa Rica. J Parasitol. (2007) 93:710–1. 10.1645/GE-1120R.1 17626370

[43] Di Guardo G Proietto U Di Francesco CE Marsilio F Zaccaroni A Scaravelli D. Cerebral toxoplasmosis in striped dolphins (*Stenella coeruleoalba*) stranded along the Ligurian Sea coast of Italy. Vet Pathol. (2010) 47:245–53. 10.1177/0300985809358036 20118319

[44] Roe WD Howe L Baker EJ Burrows L Hunter SA. An atypical genotype of Toxoplasma gondii as a cause of mortality in Hector's dolphins (*Cephalorhynchus hectori*). Vet Parasitol. (2013) 192:67–74. 10.1016/j.vetpar.2012.11.001 23207018

[45] Dailey M Stroud R. Parasites and associated pathology observed in cetaceans stranded along the Oregon coast. J Wildl Dis. (1978) 14:503–11. 10.7589/0090-3558-14.4.503 105154

[46] Morimitsu T Nagai T Ide M Ishii A Koono M. Parasitogenic octavus neuropathy as a cause of mass stranding of Odontoceti. J Parasitol. (1986) 72:469–72. 10.2307/3281689 3746567

[47] O'shea TJ Homer BL Greiner EC Layton AW. Nasitrema sp.-associated encephalitis in a striped dolphin (*Stenella coeruleoalba*) stranded in the Gulf of Mexico. J Wildl Dis. (1991) 27:706–9. 10.7589/0090-3558-27.4.706 1758040

[48] Degollada E Andre M Arbelo M Fernandez A. Incidence, pathology and involvement of Nasitrema species in odontocete strandings in the Canary Islands. Vet Rec. (2002) 150:81–2. 10.1136/vr.150.3.81 11837592

[49] Martin WE Haun CK Barrows HS Cravioto H. Nematode damage to brain of striped dolphin, Lagenorhynchus obliquidens. Trans Am Microsc Soc. (1970) 89:200–5. 10.2307/3224375 5470357

[50] Perrin WF Powers JE Role of a nematode in natural mortality of spotted dolphins. J Wildl Manage. (1980) 44:960–3 10.2307/3808335

[51] Kuiken T García Hartmann M Cetacean pathology: dissection techniques tissue sampling. In: Newsletter E editors. Proceedings of the First ECS Workshop on Cetacean Pathology. Leiden: European Cetacean Society (1993), p. 1–41.

[52] Ijsseldijk LL Brownlow AC Mazzariol SE European Best Practice on Cetacean Post-Mortem Investigation and Tissue Sampling. OSF Preprints. Joint ASCOBANS and ACCOBAMS Document Charlottesville, VA (2019).

[53] Reidenberg JS Laitman JT. Cetacean Prenatal Development. In: Perrin WF Würsig B Thewissen JGM editors. Encyclopedia of Marine Mammals, 2nd Ed. Amsterdam: Academic Press (2009), p. 228–9. 10.1016/B978-0-12-373553-9.00014-6

[54] Geraci JR Lounsbury VJ Specimen data collection. In: Geraci JR Lounsbury VL Marine Mammals Ashore: A Field Guide for Strandings. Editors. Second Edition ed. Galveston, TX: Texas A&M University Sea Grant College Program (2005). p.182–4.

[55] Joblon MJ Pokras MA Morse B Harry CT Rose KS Sharp SM Body condition scoring system for delphinids based on short-beaked common dolphins (*Delphinus delphis*). J Mar Anim Ecol. (2014) 7:5–13.

[56] Fix AS Garman RH. Practical aspects of neuropathology: a technical guide for working with the nervous system. Toxicol Pathol. (2000) 28:122–31. 10.1177/019262330002800115 10668998

[57] Sacchini S Arbelo M Bombardi C Fernández A Cozzi B Bernaldo De Quirós Y. Locus coeruleus complex of the family Delphinidae. Sci Rep. (2018) 8:5486. 10.1038/s41598-018-23827-z 29615733 PMC5883054

[58] Di Francesco G Petrini A D'angelo AR Di Renzo L Luciani M Di Febo T. Immunohistochemical investigations on Brucella ceti-infected, neurobrucellosis-affected striped dolphins (*Stenella coeruleoalba*). Vet Ital. (2019) 55:363–7. 10.12834/VetIt.1920.10224.2 31955559

[59] Sacristan C Carballo M Munoz MJ Belliere EN Neves E Nogal V. Diagnosis of cetacean morbillivirus: a sensitive one step real time RT fast-PCR method based on SYBR(®) green. J Virol Methods. (2015) 226:25–30. 10.1016/j.jviromet.2015.10.002 26454114

[60] VanDevanter DR Warrener P Bennett L Schultz ER Coulter S Garber RL. Detection and analysis of diverse herpesviral species by consensus primer PCR. J Clin Microbiol. (1996) 34:1666–71. 10.1128/JCM.34.7.1666-1671.1996 8784566 PMC229091

[61] Wu Q Mcfee WE Goldstein T Tiller RV Schwacke L. Real-time PCR assays for detection of Brucella spp. and the identification of genotype ST27 in bottlenose dolphins (*Tursiops truncatus*). J Microbiol Methods. (2014) 100:99–104. 10.1016/j.mimet.2014.03.001 24632518

[62] Baily GG Krahn JB Drasar BS Stoker NG. Detection of *Brucella melitensis* and *Brucella abortus* by DNA amplification. J Trop Med Hyg. (1992) 95:271–5. 1495123

[63] Probert WS Schrader KN Khuong NY Bystrom SL Graves MH. Real-time multiplex PCR assay for detection of Brucella spp., *B. abortus*, and *B. melitensis. J Clin Microbiol*. (2004) 42:1290–3. 10.1128/JCM.42.3.1290-1293.2004 15004098 PMC356861

[64] Edvinsson B Lappalainen M Evengard B. Real-time PCR targeting a 529-bp repeat element for diagnosis of toxoplasmosis. Clin Microbiol Infect. (2006) 12:131–6. 10.1111/j.1469-0691.2005.01332.x 16441450

[65] Diaz-Delgado J Groch KR Sierra E Sacchini S Zucca D Quesada-Canales O. Comparative histopathologic and viral immunohistochemical studies on CeMV infection among Western Mediterranean, Northeast-Central, and Southwestern Atlantic cetaceans. PLoS ONE. (2019) 14:e0213363. 10.1371/journal.pone.0213363 30893365 PMC6426187

[66] Sierra E Zucca D Arbelo M Garcia-Alvarez N Andrada M Deniz S. Fatal systemic morbillivirus infection in bottlenose dolphin, Canary Islands, Spain. Emerg Infect Dis. (2014) 20:269–71. 10.3201/eid2002.131463 24447792 PMC3901504

[67] Sierra E Fernández A Zucca D Câmara N Felipe-Jiménez I Suárez-Santana C. (2018). Morbillivirus infection in Risso's dolphin Grampus griseus: a phylogenetic and pathological study of cases from the Canary Islands. Dis Aquat Organ. 129, 165–74. 30154276 10.3354/dao03248

[68] Sierra E Fernández A Suárez-Santana C Xuriach A Zucca D Bernaldo de Quirós Y Morbillivirus and Pilot Whale Deaths, Canary Islands, Spain, 2015. Emerg Infect Dis J. (2016) 22 10.3201/eid2204.150954 PMC4806956 26982571

[69] Arbelo M Belliere EN Sierra E Sacchinni S Esperon F Andrada M. Herpes virus infection associated with interstitial nephritis in a beaked whale (*Mesoplodon densirostris*). BMC Vet Res. (2012) 8:243. 10.1186/1746-6148-8-243 23237059 PMC3577509

[70] Bouza E Garcia De La Torre M Parras F Guerrero A Rodriguez-Creixems M Gobernado J. Brucellar meningitis. Rev Infect Dis. (1987) 9:810–22. 10.1093/clinids/9.4.810 3326128

[71] Turel O Sanli K Hatipoglu N Aydogmus C Hatipoglu H Siraneci R. Acute meningoencephalitis due to Brucella: case report and review of neurobrucellosis in children. Turk J Pediatr. (2010) 52:426–9. 21043393

[72] Ceran N Turkoglu R Erdem I Inan A Engin D Tireli H. Neurobrucellosis: clinical, diagnostic, therapeutic features and outcome. Unusual clinical presentations in an endemic region. Braz J Infect Dis. (2011) 15:52–9. 10.1016/S1413-8670(11)70140-4 21412590

[73] Hatami H Hatami M Soori H Janbakhsh AR Mansouri F. Epidemiological, clinical, and laboratory features of brucellar meningitis. Arch Iran Med. (2010) 13:486–91. 21039003

[74] Rossi M Tascini C Carannante N Di Caprio G Sofia S Iacobello C. Neurobrucellosis: diagnostic and clinical management of an atypical case. New Microbiol. (2018) 41:165–7. 29384559

[75] Cowan DF. Pathology of the pilot whale. Globicephala melaena A comparative survey. Arch Pathol. (1966) 82:178–89. 5949961

[76] Shieh W-J Zaki SR Advanced pathology techniques for detecting emerging infectious disease pathogens. In: Tang YW Stratton CW editors. Advanced Techniques in Diagnostic Microbiology. Boston, MA: Springer US (2013). p. 873–90. 10.1007/978-1-4614-3970-7_45

