# Supplementary material for: Histopathological Differential Diagnosis of Meningoencephalitis in Cetaceans: Morbillivirus, Herpesvirus, Toxoplasma gondii, Brucella sp., and Nasitrema sp
Source: Front Vet Sci. 2020 Sep 30;7:650. doi: 10.3389/fvets.2020.00650 (PMC7554640; doi:10.3389/fvets.2020.00650)
Supplement: Supplementary file 1 [file Table_1.docx]

Table 1. Epidemiologic and biologic data on stranded cetaceans under study.

| **Case No.** | **Lab. Ref.** | **Species** | **Sex** | **Age** | **ST** | **BC** | **DC** | **SD** | **SL** |
| --- | --- | --- | --- | --- | --- | --- | --- | --- | --- |
| 1 | I-082/01 (CET 123) | *Stenella frontalis* | M | Adult | P | Good | Moderate autolysis | 26/03/2001 | Gran Canaria (C.I.) |
| 2 | I-086/01 (CET 124) | *Tursiops truncatus* | M | Adult | P | Poor | Fresh | 11/04/2001 | Tenerife (C.I.) |
| 3 | I-381/01 (CET 152) | *Stenella frontalis* | M | Adult | P | Good | Advanced autolysis | 27/12/2001 | Tenerife (C.I.) |
| 4 | I-154/02 (CET 195) | *Stenella coeruleoalba* | M | Adult | A | Good | Very fresh | 13/11/2002 | Gran Canaria (C.I.) |
| 5 | I-130/04 (CET 260) | *Stenella coeruleoalba* | F | Juvenile | A | Good | Fresh | 27/06/2004 | Fuerteventura (C.I.) |
| 6 | I-225/05 (CET 305) | *Tursiops truncatus* | F | Subadult | A | Moderate | Very fresh | 18/07/2005 | Lanzarote (C.I.) |
| 7 | I-265/05 (CET 308) | *Stenella coeruleoalba* | F | Juvenile | A | Good | Fresh | 16/08/2005 | Gran Canaria (C.I.) |
| 8 | I-022/07 (CET 364) | *Delphinus delphis* | M | Adult | P | Poor | Moderate autolysis | 20/01/2007 | Gran Canaria (C.I.) |
| 9 | I-071/07 (CET 373) | *Delphinus delphis* | F | Adult | A | Moderate | Fresh | 26/03/2007 | Lanzarote (C.I.) |
| 10 | I-091/07 (CET 380) | *Stenella coeruleoalba* | M | Subadult | P | Poor | Fresh | 16/04/2007 | Tenerife (C.I.) |
|  | I-154/07 (CET 384) | *Stenella frontalis* | M | Adult |  | Good | NA | NA | Gran Canaria (C.I.) |
| 11 | I-137/08 (CET 409) | *Stenella coeruleoalba* | F | Subadult | P | Poor | Moderate autolysis | 05/02/2008 | Fuerteventura (C.I.) |
| 12 | I-149/08 (CET 431) | *Grampus griseus* | M | Juvenile | A | Poor | Fresh | 21/04/2008 | Tenerife (C.I.) |
| 13 | I-063/09 (CET 475) | *Stenella frontalis* | F | Calf | P | Moderate | Moderate autolysis | 10/01/2009 | Tenerife (C.I.) |
| 14 | I-007/09 (CET 476) | *Stenella coeruleoalba* | F | Adult | P | Moderate | Fresh | 22/01/2009 | Fuerteventura (C.I.) |
| 15 | I-119/10 (CET 515) | *Stenella frontalis* | M | Adult | A | Good | Fresh | 26/02/2010 | Fuerteventura (C.I.) |
| 16 | I-125/10 (CET 522) | *Stenella frontalis* | M | Adult | P | Moderate | Moderate autolysis | 24/03/2010 | Lanzarote (C.I.) |
| 17 | I-123/10 (CET 530) | *Stenella frontalis* | F | Adult | P | Moderate | Fresh | 13/04/2010 | Tenerife (C.I.) |
| 18 | I-033/11 (CET 554) | *Stenella coeruleoalba* | F | Adult | A | Moderate | Fresh | 21/01/2011 | Gran Canaria (C.I.) |
| 19 | I-014/11 (CET 558) | *Stenella coeruleoalba* | F | Adult | P | Good | Fresh | 10/02/2011 | Lanzarote (C.I.) |
| 20 | I-083/11 (CET 564) | *Tursiops truncatus* | M | Adult | P | Good | Fresh | 22/03/2011 | Lanzarote (C.I.) |
| 21 | I-145/11 (CET 574) | *Stenella coeruleoalba* | M | Subadult | A | Poor | Fresh | 01/05/2011 | Gran Canaria (C.I.) |
| 22 | I-158/11 (CET 575) | *Globicephala macrorhynchus* | M | Calf | P | Very poor | Moderate autolysis | 10/05/2011 | Fuerteventura (C.I.) |
| 23 | I-229/11 (CET 583) | *Globicephala macrorhynchus* | M | Juvenile | A | Moderate | Moderate autolysis | 23/07/2011 | Tenerife (C.I.) |
| 24 | I-379/11 (CET 594) | *Globicephala macrorhynchus* | M | Subadult | P | Very poor | Fresh | 02/11/2011 | Tenerife (C.I.) |
| 25 | I-065/12 (CET 614) | *Stenella coeruleoalba* | M | Subadult | P | Good | Fresh | 28/04/2012 | Lanzarote (C.I.) |
| 26 | I-071/12 (CET 618) | *Stenella frontalis* | M | Subadult | P | Moderate | Advanced autolysis | 12/05/2012 | Tenerife (C.I.) |
| 27 | I-067/13 (CET 666) | *Globicephala macrorhynchus* | M | Subadult | A | Moderate | Fresh | 15/05/2013 | Lanzarote (C.I.) |
| 28 | I-289/13 (NA) | *Stenella coeruleoalba* | M | Calf | P | Poor | Moderate autolysis | 31/08/2013 | Cádiz (Andalusia) |
| 29 | I-151/14 (CET 717) | *Stenella coeruleoalba* | M | Juvenile | A | Poor | Very fresh | 21/05/2014 | Gran Canaria (C.I.) |
| 30 | I-317/14 (CET 732) | *Stenella coeruleoalba* | F | Calf | P | Moderate | Fresh | 11/11/2014 | Gran Canaria (C.I.) |
| 31 | I-280/15 (CET 758) | *Globicephala macrorhynchus* | M | Calf | F | Moderate | Fresh | 15/05/2015 | Fuerteventura (C.I.) |
| 32 | I-416/15 (CET 772) | *Stenella coeruleoalba* | F | Adult | P | Good | Fresh | 21/08/2015 | Lanzarote (C.I.) |
| 33 | I-08/16 (NA) | *Ziphius cavirostris* | M | Adult | ND | ND | Moderate autolysis | 02/12/2015 | Huelva (Andalusia) |
| 34 | I-287/16 (CET 798) | *Stenella frontalis* | M | Adult | F | Moderate | Fresh | 08/04/2016 | Tenerife (C.I.) |
| 35 | I-907/16 (CET 810) | *Delphinus delphis* | F | Juvenile | A | Very poor | Fresh | 03/07/2016 | Tenerife (C.I.) |
| 36 | I-167/17 (CET 854) | *Stenella frontalis* | F | Juvenile | P | Poor | Moderate autolysis | 15/05/2017 | Tenerife (C.I.) |
| 37 | SA038/18 (CET 884) | *Stenella frontalis* | F | Calf | P | Moderate | Fresh | 16/01/2018 | Tenerife (C.I.) |
| 38 | SA223/18 (CET 921) | *Stenella coeruleoalba* | M | Juvenile | P | Poor | Moderate autolysis | 05/07/2018 | Gran Canaria (C.I.) |

Lab. Ref.: Laboratorial Reference; sex (F: female, M: male); ST: Stranding type (A: active, P: pasive); BC: Body Condition; DC: Decomposition Code; SD: Stranding Date; SL: Stranding Location. NA: Not Available
