# Supplementary material for: Histopathological Differential Diagnosis of Meningoencephalitis in Cetaceans: Morbillivirus, Herpesvirus, Toxoplasma gondii, Brucella sp., and Nasitrema sp
Source: Front Vet Sci. 2020 Sep 30;7:650. doi: 10.3389/fvets.2020.00650 (PMC7554640; doi:10.3389/fvets.2020.00650)
Supplement: Supplementary file 2 [file Table_2.docx]

Table 2. Results [positive, negative or non applied (-)] of molecular, immnunohistochemical, and histochemical assays performed on brain samples from animals under study.

| **Case No.** | **Ref. Lab.** | **PCR** | | | | | **IHC** | **HC** |
| --- | --- | --- | --- | --- | --- | --- | --- | --- |
|  |  | CeMV | HV | *Brucella* spp. | *T. gondii* | *Nasitrema* spp. |  |  |
| 1 | I-082/01 (CET 123) | Negative^a^ | - | Negative^a^ | Positive^b^ | - | *T. gondii* | PAS |
| 2 | I-086/01 (CET 124) | Negative^a^ | Positive | - | - | - | - | - |
| 3 | I-381/01 (CET 152) | Negative^a^ | - | Negative^a^ | Positive^b^ | - | *T. gondii* | PAS |
| 4 | I-154/02 (CET 195) | DMV^b^ | Negative | Negative^b^ | Negative^a^ | - | CDV | - |
| 5 | I-130/04 (CET 260) | Negative^c^ | Negative | Positive^a, b^ | - | - | *Brucella* sp. | - |
| 6 | I-225/05 (CET 305) | DMV^a, c^ | Negative | Positive^a^ | - | - | CDV/*Brucella* sp. | - |
| 7 | I-265/05 (CET 308) | DMV^a, c^ | Negative | Negative^a^ | - | - | CDV | - |
| 8 | I-022/07 (CET 364) | Negative^c^ | Negative | Positive^a^ | - | - | - | - |
| 9 | I-071/07 (CET 373) | Negative^b^ | Negative | Negative^b^ | Negative^a^ | - | CDV | - |
| 10 | I-091/07 (CET 380) | DMV^b^ | Positive | Negative^b^ | Negative^a^ | - | CDV | - |
| 11 | I-137/08 (CET 409) | DMV^b^ | Negative | Negative^b^ | Negative^a^ | - | CDV | - |
| 12 | I-149/08 (CET 431) | DMV^a^ | Negative | Negative^a^ | - | - | CDV | - |
| 13 | I-063/09 (CET 475) | Negative^a^ | - | - | Positive^b^ | - | *T. gondii* | PAS |
| 14 | I-007/09 (CET 476) | DMV^b^ | Negative | Negative^b^ | Negative^a^ | - | CDV | - |
| 15 | I-119/10 (CET 515) | Negative^a^ | - | Negative^a^ | Positive^b^ | - | *T. gondii* | PAS |
| 16 | I-125/10 (CET 522) | Negative^a^ | - | Negative^a^ | Positive^b^ | - | *T. gondii* | PAS |
| 17 | I-123/10 (CET 530) | Negative^a^ | Negative | Negative^a^ | Positive^b^ | - | *T. gondii* | PAS |
| 18 | I-033/11 (CET 554) | Negative^b^ | Positive | Negative^b^ | Negative^a^ | - | - | - |
| 19 | I-014/11 (CET 558) | DMV^b^ | Negative | Negative^b^ | Negative^a^ | - | CDV | - |
| 20 | I-083/11 (CET 564) | Negative^a^ | Negative | Negative^a^ | - | Positive | - | - |
| 21 | I-145/11 (CET 574) | Negative^b^ | Positive | Negative^b^ | Negative^a^ | - | - | - |
| 22 | I-158/11 (CET 575) | Negative^c^ | - | Positive^a^ | - | - | - | - |
| 23 | I-229/11 (CET 583) | PWMV^c^ | - | - | - | - | CDV | - |
| 24 | I-379/11 (CET 594) | PWMV^c^ | - | - | - | - | CDV | - |
| 25 | I-065/12 (CET 614) | DMV^a^ | - | - | - | - | CDV | - |
| 26 | I-071/12 (CET 618) | Negative^c^ | - | Positive^a^ | - | - | - | - |
| 27 | I-067/13 (CET 666) | PWMV^a, c^ | - | - | - | - | CDV | - |
| 28 | I-289/13 (NA) | Negative^a, c^ | Positive | - | - | - | - | - |
| 29 | I-151/14 (CET 717) | Negative^a^ | Positive | - | - | - | - | - |
| 30 | I-317/14 (CET 732) | Negative^a, c^ | Negative | Positive^a^ | - | - | - | - |
| 31 | I-280/15 (CET 758) | PWMV^a, c^ | Negative | - | Negative^b^ | - | CDV | PAS/Grocott |
| 32 | I-416/15 (CET 772) | Negative^a, c^ | Positive | - | - | - | - | - |
| 33 | I-08/16 (NA) | Negative^a^ | Positive | - | - | - | - | - |
| 34 | I-287/16 (CET 798) | Negative^c^ | Positive | - | - | - | - | - |
| 35 | I-907/16 (CET 810) | DMV^a^ | Positive | - | - | - | CDV | - |
| 36 | I-167/17 (CET 854) | Negative^c^ | Positive | Negative^a^ | Negative^b^ | - | - | - |
| 37 | SA038/18 (CET 884) | Negative^c^ | Positive | Positive^a^ | - | - | - | - |
| 38 | SA223/18 (CET 921) | DMV^c^ | Positive | - | - | - | CDV | - |

Ref. Lab.: Laboratorial Reference; CeMV: Cetacean Morbillivirus (DMV: Dolphin Morbillivirus, PWMV: Pilot Whale Morbillivirus); HV: Herpesvirus; *T. gondii:* *Toxoplasma gondii*; IHC: Immunohistochemistry; HC: Histochemistry; PCR: Polymerasa Chain Reaction methods:

- CeMV: a, one-step RT-PCR of a 426-bp conserved region of the phosphoprotein (P) gene; b, RT-PCR using nested primers also targeted at the P gene; c, one-step real-time RT-PCR to detect sequences in a conserved region (192 bp) of the fusion protein gene.
- HV: conventional nested PCR using degenerate primers designed to amplify a region of the DNA polymerase gene
- *Brucella* spp.: a, quantitative duplex-PCR amplifying a 150 bp fragment of the IS711 gene for the detection of Brucella at the genus level and the identification of genotype ST27; b, PCR using primers amplifying a 223-bp fragment of the bcsp31 gene.
- *T. gondii*: a, real-time PCR targeting a 529-bp repeat element of *T. gondii*; b, real-time PCR based on a 163 bp sequence within the *T. gondii* small subunit ribosomal RNA (18SrRNA)
- *Nasitrema* spp.: real time PCR based upon the partial NADH dehydrogenase, subunit 3 gene of *Nasitrema delphini*.
